# Chemoenzymatic Synthesis and Chemical Recycling of Poly(ester-urethane)s

**DOI:** 10.3390/ijms12095490

**Published:** 2011-08-29

**Authors:** Hiroto Hayashi, Yoshio Yanagishita, Shuichi Matsumura

**Affiliations:** Department of Applied Chemistry, Faculty of Science and Technology, Keio University, 3-14-1 Hiyoshi, Kohoku-ku, Yokohama 223-8522, Japan

**Keywords:** enzyme-catalyzed polymerization, chemical recycling, green polymer, polyurethane, poly(ester-urethane)

## Abstract

Novel poly(ester-urethane)s were prepared by a synthetic route using a lipase that avoids the use of hazardous diisocyanate. The urethane linkage was formed by the reaction of phenyl carbonate with amino acids and amino alcohols that produced urethane-containing diacids and hydroxy acids, respectively. The urethane diacid underwent polymerization with polyethylene glycol and α,ω-alkanediols and also the urethane-containing hydroxy acid monomer was polymerized by the lipase to produce high-molecular-weight poly(ester-urethane)s. The periodic introduction of ester linkages into the polyurethane chain by the lipase-catalyzed polymerization afforded chemically recyclable points. They were readily depolymerized in the presence of lipase into cyclic oligomers, which were readily repolymerized in the presence of the same enzyme. Due to the symmetrical structure of the polymers, poly(ester-urethane)s synthesized in this study showed higher *T*_m_, Young’s modulus and tensile strength values.

## 1. Introduction

Polyurethane is widely used in many products, such as the manufacture of plastic foams, sponges, paints, coatings and fibers. Conventional polyurethane is mostly produced from polyols and toxic diisocyanate derived from the even more toxic phosgene. Diisocyanate- and phosgene-free methods for the production of the next generation of polyurethanes, as well as fast chemical recyclability and high performance, must be addressed. Recently, poly(ester-uretane)s containing biobased soft segments, such as polylactide diol and castor oil, have been investigated. However, the urethane moiety as a hard segment is created using toxic diisocyanates. Furthermore, diisocyanates are very reactive and produce complex polyurethane structures, such as branching, allophanates and biurets in addition to the main urethane linkage. With respect to biomaterials, chemical recycling and biodegradabilities, a more unimodal polyurethane structure will be needed. In order to establish a sustainable chemical society, commodity polymers should have facile and complete chemical recyclabilities.

There have been some attempts to develop diisocyanate- and phosgene-free methods for the production of polyurethanes, such as the reaction of cyclic or phenyl carbonates and amines to form a urethane linkage [1–6]. The industrial production of diphenyl carbonate from carbon dioxide and methanol with subsequent treatment by phenol has been established as a green process. Thus, the direct condensation of diphenyl carbonate and an amine may become a green process for production of the urethane linkage.

For the chemical recycling of polyurethane, hydrolysis [7,8], glycolysis [9,10] and aminolysis [11] have been reported for recovering polyols and amines. However, the addition of diisocyanates is required for the regeneration of the polyurethane. For green and sustainable chemistry, repetitive chemical recycling of polymeric materials between the polymer and monomer forms is an ideal process. Thus, enzyme-catalyzed polymerization and degradation may become a versatile method for the production of green and sustainable polyurethanes. Namely, the key advantage of using a lipase for the production of the polymer is the reversible polymerization-depolymerization reaction that enables the chemical recycling process. Polymer chains that contain enzymatically hydrolyzable moieties, such as ester and carbonate linkages, can be cleaved by a lipase in a dilute organic solvent to produce a repolymerizable cyclic oligomer that can be recycled [12].

We initially reported the chemoenzymatic preparation of poly(diester-urethane) [13] and poly(diester-diurethane) [14]. However, the former had a relatively low *T*_m_ of around 40 °C [13], while the latter was relatively hard and brittle [14]. In this report, poly(ester-urethane)s having moderate elongation properties and *T*_m_s of around 100 °C were designed and synthesized by green processes. Their thermal and mechanical properties and chemical recyclabilities by lipase were reported.

## 2. Experimental Section

### 2.1. Materials

Butane-1,4-diol, octane-1,8-diol, decane-1,10-diol, dodecane-1,12-diol, diphenyl carbonate, 12-amino-1-dodecanol and 1,2-dimethoxybenzene were purchased from Tokyo Kasei Kogyo Co., Inc. (Tokyo, Japan). Hexaethylene glycol, octaethylene glycol and anisole (anhydrous) were purchased from Aldrich Chem. Co., Inc. (Milwaukee, WI, USA). 12-Aminododecanoic acid and toluene (anhydrous) were purchased from Wako Chemical Co., Inc (Tokyo, Japan). Immobilized lipase from *Candida antarctica* was kindly supplied by Novozymes Japan Ltd. (Chiba, Japan); CALB: Novozym 435, a lipase (lipase B) from *C. antarctica* produced by submerged fermentation of a genetically modified *Aspergillus oryzae* microorganism and absorbed on a macroporous acrylic resin (10,000 propyl laurate units per gram; lipase activity based on ester synthesis). The enzyme was dried under vacuum over P_2_O_5_ at 25 °C for 6 h. Thermally deactivated CALB was prepared by heating the enzyme for 15 min in steam using an autoclave at 120 °C and then freeze-drying under vacuum.

### 2.2. Measurements

The weight-average (*M*_w_) and number-average (*M*_n_) molecular weight and the molecular weight distribution (*M*_w_/*M*_n_) of the polymer were measured by means of size exclusion chromatography (SEC) using SEC columns (Shodex K-805L + K-800D, Showa Denko Co., Ltd., Tokyo, Japan) with a refractive index detector ; chloroform was used as the eluent at 1.0 mL·min^−1^. The *M*_w_, *M*_n_ and *M*_w_/*M*_n_ values of the oligomers were also measured by means of SEC (Shodex K-802 + K-800D, Showa Denko Co., Ltd., Tokyo, Japan) under the same conditions. The SEC system was calibrated with polystyrene standards of narrow molecular weight distribution. The molecular weight of oligomers was also measured by the matrix-assisted laser desorption ionization time-of-flight (MALDI-TOF) mass spectra using a Bruker Ultraflex mass spectrometer equipped with a nitrogen laser. The detection was performed in the reflection mode, a cobalt matrix was used for oligomers, sodium bromide was used as the cation source, and positive ionization was used. Supercritical carbon dioxide fluid (scCO_2_) chromatography (SFC) was used for the fractionation of the oligomer mixture, using a JASCO SFC-201 chromatograph equipped with a 4.6 mm i.d. × 250 mm preparative column packed with silica gel (SFCpak SIL-5, JASCO Ltd., Tokyo, Japan), using scCO_2_ as the mobile phase and ethanol as a modifier. The scCO_2_ pressure was controlled at 25 MPa and the column temperature was maintained at 70 °C. Chromatograms were recorded using a UV detector operating at a wavelength of 215 nm. ^1^H NMR spectra were recorded on a Varian 300 Fourier transform spectrometer (Varian Inc., CA, USA) operating at 300 MHz, and ^13^C NMR spectra were recorded on a ECA-500 Fourier transform spectrometer (JEOL Ltd., Tokyo, Japan) operating at 125 MHz.

The glass-transition temperatures (*T*_g_), crystallization temperatures (*T*_c_) and melting temperatures (*T*_m_) of the polymers were determined by differential scanning calorimetry (DSC-60, Shimadzu Co., Kyoto, Japan). The measurements were made with a sample (3–5 mg) on a DSC plate. The polymer samples were heated at the rate of 10 °C·min^−1^ from 30 °C to 150 °C (first scan), rapidly cooled to −50 °C at the rate of −20 °C·min^−1^, and then scanned at the same heating rate of 10 °C over the temperature range of −50 °C to 150 °C (second scan). The Young’s modulus of film samples produced by the hot-press method was measured using an Autograph instrument (Shimadzu Co., Kyoto, Japan) at the cross-head speed of 30 mm·min^−1^. The static water contact angle of film samples produced by the solvent-cast method was measured using a contact angle meter (DMs-400, Kyowa Interface Science Co., Ltd., Saitama, Japan).

### 2.3. Preparation of Diol-Diacid-Type Poly(ester-ether-urethane) (PEEU) and Poly(ester-urethane) (PEU)

Preparation of PEEU and PEU are outlined in Scheme I.

#### Preparation of tetramethylene bis(phenyl carbonate) (**1**)

**1** was prepared by the reaction of diphenyl carbonate with butane-1,4-diol using CALB as a catalyst. In a typical procedure, diphenyl carbonate (3.64 g; 17.0 mmol) and butane-1,4-diol (76.4 mg; 0.85 mmol) were dissolved in toluene (12 mL). CALB (364 mg: 10 wt% relative to diphenyl carbonate) was then added, and the mixture was stirred under a nitrogen atmosphere at 70 °C for 24 h. The reaction mixture was diluted with chloroform and the immobilized enzyme was removed by filtration. The crude product was purified by column chromatography (silica gel 60N, 150 g) using chloroform as eluent to obtain **1** in 44.1% yield (*R*_f_ = 0.40).

^1^H NMR (300 MHz, DMSO-d_6_): δ = 1.79 (m, 4H, -CH_2_CH_2_OCOO-), 4.26 (m, 4H, -CH_2_CH_2_OCOO-), 7.22–7.46 (m, 10H, C_6_H_5_OCOO-). ^13^C NMR (125 MHz, DMSO-d_6_): δ = 24.5 (-CH_2_CH_2_OCOO-), 68.0 (-CH_2_CH_2_OCOO-), 121.3 (aromatic C_2_), 126.1 (aromatic C_4_), 129.6 (aromatic C_3_), 150.8 (aromatic C_1_), 153.1 (-OCOO-). Elemental analysis (%) calcd for C_18_H_18_O_6_ (330.33): C, 65.45; H, 5.49; found C, 65.42; H, 5.53.

#### Preparation of diurethane-containg dicarboxylic acid (**2**)

**2** was prepared by the reaction of **1** with sodium 12-aminododecanoate. In a typical procedure, 12-aminododecanoic acid (2.21 g; 10.3 mmol) was saponified by sodium hydroxide (410.8 mg; 10.3 mmol) in methanol (35 mL) at 60 °C for 1 h with stirring. The solvent was then evaporated under reduced pressure to afford sodium 12-aminododecanoate quantitatively. **1** (165.1 mg; 0.50 mmol) and sodium 12-aminododecanoate (296.7 mg; 1.25 mmol) was dissolved in chloroform/methanol = 1/1 (v/v) (1 mL), and the mixture was stirred under a nitrogen atmosphere at 50 °C for 45 min. The solvent was then evaporated under reduced pressure, and residue was acidified with 1*_N_* HCl to pH 1–2. The crude product was further purified by dissolving in a small amount of chloroform/methanol = 1/1 (v/v) and reprecipitation using cold ethanol to afford **2** in 66.7% yield.

^1^H NMR (300 MHz, DMSO-d_6_): δ = 1.23 (m, 28H, -(CH_2_)_7_-(CH_2_)_2_-NH-), 1.36 (m, 4H, -CH_2_CH_2_NH-), 1.47 (m, 4H, HOOCCH_2_CH_2_CH_2_-), 1.55 (m, 4H, -NHCOOCH_2_-(CH_2_)_2_-CH_2_OCOO-), 2.18 (t, 4H, HOOCCH_2_CH_2_-, J = 7.5 Hz), 2.93 (td, 4H, -CH_2_NH-, J = 6.6, 6.6 Hz), 3.92 (m, 4H, -NHCOOCH_2_CH_2_-), 6.73–7.06 (br, 2H, -NHCOO-). ^13^C NMR (125 MHz, DMSO-d_6_): δ = 24.5 (HOOCCH_2_CH_2_-), 25.4 (-NHCOOCH_2_CH_2_-), 26.3 (-CH_2_-(CH_2_)_2_-NH-), 28.6 (HOOC-(CH_2_)_2_-CH_2_-), 28.8 (HOOC-(CH_2_)_3_-CH_2_-, -CH_2_-(CH_2_)_3_-NH-), 29.0 (HOOC-(CH_2_)_4_-(CH_2_)_3_-), 29.4 (-CH_2_CH_2_NH-), 33.7 (HOOCCH_2_-), 40.2 (-CH_2_NH-), 63.1 (-NHCOOCH_2_-), 156.2 (-NHCOOCH_2_-), 174.5 (HOOCCH_2_-). Elemental analysis (%) calcd for C_30_H_56_N_2_O_8_ (572.77): C, 62.91; H, 9.85; N, 4.89; found C, 62.72; H, 9.82; N, 4.84.

#### Preparation of PEEU(6) by direct-polycondensation of **2** and hexaethylene glycol

The lipase-catalyzed polymerization was carried in a vial equipped with a small column packed with molecular sieves 4A placed at the top of the vial. In a typical procedure, **2** (11.4 mg; 0.02 mmol) and hexaethylene glycol (5.6 mg; 0.02 mmol) were dissolved in 1,2-dimethoxybenzene (0.057 mL), CALB (6.8 mg: 60 wt% relative to **2**) was added, and the mixture was stirred under a nitrogen atmosphere at 100 °C for 48 h. The reaction mixture was dissolved in chloroform (5 mL) at 60 °C and the insoluble enzyme was removed by filtration. The filtrates were then evaporated under reduced pressure to obtain the crude polymer, which was further purified by dissolving in a small amount of DMF and reprecipitaion using methanol to afford PEEU(6) in 78.9% yield (*M*_w_ = 136,000 g·mol^−1^).

^1^H NMR (300 MHz, CDCl_3_): δ = 1.28 (m, 28H, -(CH_2_)_7_-(CH_2_)_2_-NH-), 1.48 (m, 4H, -CH_2_CH_2_NH-), 1.63 (m, 4H, -OOCCH_2_CH_2_CH_2_-), 1.69 (m, 4H, -NHCOOCH_2_-(CH_2_)_2_-CH_2_COO-), 2.33 (t, 4H, -OOCCH_2_CH_2_-, J = 7.5 Hz), 3.16 (td, 4H, -CH_2_NH-, J = 6.6, 6.6 Hz), 3.66 (m, 16H, -OCH_2_CH_2_O- (CH_2_CH_2_O)_4_-CH_2_CH_2_O-), 3.70 (t, 4H, -OCH_2_CH_2_O-(CH_2_CH_2_O)_4_-CH_2_CH_2_O-, J = 4.8 Hz), 4.09 (m, 4H, -NHCOOCH_2_CH_2_-), 4.23 (t, 4H, -OCH_2_CH_2_O-(CH_2_CH_2_O)_4_-CH_2_CH_2_O-, J = 4.8 Hz), 4.47–4.72 (br, 2H, -NHCOO-). In a similar procedure a series of PEEU(x)s was prepared.

#### Preparation of PEU(12) by direct-polycondensation of **2** and 1,12-dodecanediol

The lipase-catalyzed polymerization was carried out in a same procedure as described for PEEU, except that an alkanediol was used instead of polyethylene glycol. The crude polymer was purified by dissolving in a small amount of chloroform and reprecipitaion using methanol/hexane = 1/1 (v/v) to afford PEU(12) in 86.3 % yield (*M*_w_ = 101,000 g·mol^−1^).

^1^H NMR (300 MHz, CDCl_3_): δ = 1.27 (m, 44H, -(CH_2_)_7_-(CH_2_)_2_-NH-, -O-(CH_2_)_2_-(CH_2_)_8_-), 1.48 (m, 4H, -CH_2_CH_2_NH-), 1.61 (m, 8H, -OOCCH_2_CH_2_CH_2_-, -O-CH_2_CH_2_CH_2_-), 1.67 (m, 4H, -NHCOOCH_2_- (CH_2_)_2_-CH_2_COO-), 2.29 (t, 4H, -OOCCH_2_CH_2_-, J = 7.5 Hz), 3.15 (td, 4H, -CH_2_NH-, J = 6.6, 6.6 Hz), 4.05 (m, 8H, -NHCOOCH_2_CH_2_-, -OCH_2_CH_2_-), 4.49-4.71 (br, 2H, -NHCOO-). In a similar procedure a series of PEU(y)s was prepared. ^1^H NMR spectra of PEEU(8), PEU(8) and PEU(10) are shown in Appendix.

### 2.4. Preparation of Hydroxy Acid Type Poly(ester-diurethane) (PEDU)

Preparation of PEDU is outlined in Scheme II.

#### Preparation of urethane-containing hydroxy phenyl carbonate (**3**)

12-Amino-1-dodecanol (604.1 mg; 3.0 mmol) was first dissolved in chloroform (6 mL), **1** (991.0 mg; 3.0 mmol) was added, and the mixture was stirred under a nitrogen atmosphere at 40 °C for 24 h. After the reaction, the crude product was purified by column chromatography (silica gel 63.9 g), using chloroform/ethyl acetate = 1/5 (v/v) to obtain **3** in 50.1 % yield (*R*_f_ = 0.29).

^1^H NMR (300 MHz, DMSO-d_6_): δ = 1.23 (m, 16H, -(CH_2_)_8_-(CH_2_)_2_-NH-), 1.37 (m, 4H, OHCH_2_CH_2_CH_2_-, -CH_2_CH_2_NH-), 1.66 (m, 4H, -NHCOOCH_2_-(CH_2_)_2_-CH_2_OCOO-), 2.94 (td, 2H, -CH_2_NH-, J = 6.5, 6.5 Hz), 3.36 (m, 2H, HOCH_2_CH_2_-), 3.96 (t, 2H, -NHCOOCH_2_CH_2_-, J = 6.1 Hz), 4.22 (t, 2H, -CH_2_CH_2_OCOO-, J = 6.1 Hz), 4.31 (t, H, HOCH_2_-, J = 5.1 Hz), 6.65–7.10 (br, H, -NHCOO-), 7.18–7.47 (-OCOOC_6_H_5_-). Elemental analysis (%) calcd for C_24_H_39_NO_6_ (437.57): C, 65.88; H, 8.98; N, 3.20; found C, 65.95; H, 9.10; N, 3.19.

#### Preparation of diurethane-containing hydroxy acid (**4**)

Sodium 12-aminododecanoate (284.4 mg; 1.20 mmol) was dissolved in ethylene glycol (0.5 mL), **3** (262.6 mg; 0.60 mmol) was added, and the mixture was stirred under a nitrogen atmosphere at 100 °C for 1 h. The reaction mixture was acidified with 1*_N_* HCl to pH = 1–2 and the insoluble product was filtrated. The solid was washed with distilled water, methanol, and chloroform, and then dissolved in chloroform/methanol = 1/1 (v/v). The solvent was then evaporated under reduced pressure to obtain **4** in 58.8 % yield.

^1^H NMR (300 MHz, DMSO-d_6_): δ = 1.23 (m, 30H, HO-(CH_2_)_2_-(CH_2_)_8_-(CH_2_)_2_-NH-, HOOC-(CH_2_)_2_-(CH_2_)_7_-(CH_2_)_2_-NH-), 1.36 (m, 6H, HOCH_2_CH_2_CH_2_-, -CH_2_CH_2_NH-), 1.47 (m, 2H, HOOCCH_2_CH_2_CH_2_-), 1.54 (m, 4H, -NHCOOCH_2_-(CH_2_)_2_-CH_2_OCOO-), 2.16 (t, 2H, HOOCCH_2_CH_2_-, J = 7.5 Hz), 2.92 (td, 4H, -CH_2_NH-, J = 6.5, 6.5 Hz), 3.34 (t, 2H, HOCH_2_CH_2_-, J = 4.5 Hz), 3.92 (m, 4H, -NHCOOCH_2_CH_2_-), 6.63-7.10 (br, 2H, -NHCOO-). Elemental analysis (%) calcd for C_30_H_58_N_2_O_7_ (437.57): C, 64.48; H, 10.46; N, 5.01; found C, 64.49; H, 10.58; N, 4.98.

#### Preparation of PEDU by polycondensation of **4**

In a typical procedure, **4** (40.0 mg) was dissolved in anisole (0.20 mL), CALB (16.0 mg 40 wt% relative to the monomer) was added, and the mixture was stirred in a vial equipped with a small column packed with molecular sieves 4A placed at the top of the vial under a nitrogen atmosphere at 110 °C for 48 h. The reaction mixture was dissolved in chloroform (5 mL) at 60 °C, and the insoluble enzyme was removed by filtration. The solvent was then evaporated under reduced pressure to obtain the crude polymer, which was further purified by dissolving with a small amount of chloroform and reprecipitation using DMF to afford PEDU in 90.9% yield (*M*_w_ = 109,000 g·mol^−1^).

^1^H NMR (300 MHz, CDCl_3_): δ = 1.26 (m, 30H, -O-(CH_2_)_2_-(CH_2_)_8_-(CH_2_)_2_-NH-, -OOC-(CH_2_)_2_- (CH_2_)_7_-(CH_2_)_2_-NH-), 1.45 (m, 4H, -CH_2_CH_2_COOCH_2_CH_2_-), 1.61 (m, 4H, -CH_2_CH_2_NH-), 1.67 (m, 4H, -NHCOOCH_2_-(CH_2_)_2_-CH_2_OCOO-), 2.29 (t, 2H, -OOCCH_2_CH_2_-, J = 7.5 Hz), 3.15 (td, 4H, -CH_2_NH-, J = 6.6, 6.6 Hz), 4.05 (m, 6H, -NHCOOCH_2_CH_2_-, -CH_2_COOCH_2_-), 4.40-4.82 (br, 2H, -NHCOO-).

### 2.5. Chemical Recycling of PEEU(6) and PEU(12)

#### Enzymatic degradation of PEEU(6) and PEU(12) into cyclic monomer

In a typical procedure, PEEU (6) (*M*_w_ = 90,000 g·mol^−1^, 60.0 mg) or PEU(12) (*M*_w_ = 67,000 g·mol^−1^, 60.0 mg) was dissolved in anisole (20 mL) in a round bottom flask equipped with a small column packed with molecular sieves 4A placed at the top of the flask, CALB (120.0 mg) was added, and the mixture was stirred under a nitrogen atmosphere at 110 °C for 72 h. The reaction mixture was diluted with chloroform and the insoluble enzyme was removed by filtration. The solvent was then evaporated under reduced pressure to obtain the crude degradation products, which were further purified by dissolving in a small amount of chloroform and reprecipitation using ethanol. The ethanol soluble part was collected and the solvent was then evaporated under reduced pressure. The crude oligomer was then fractionated using a preparative SFC column with scCO_2_ and ethanol as a modifier to afford the cyclic(ester-ether-urethane) (CEEU(6)) monomer or the cyclic(ester-urethane) (CEU(12)) monomer.

CEEU(6): ^1^H NMR (300 MHz, CDCl_3_): δ = 1.28 (m, 28H, -(CH_2_)_7_-(CH_2_)_2_-NH-), 1.48 (m, 4H, -CH_2_CH_2_NH-), 1.62 (m, 4H, -OOCCH_2_CH_2_CH_2_-), 1.69 (m, 4H, -NHCOOCH_2_-(CH_2_)_2_-CH_2_COO-), 2.33 (t, 4H, -OOCCH_2_CH_2_-, J = 7.5 Hz), 3.16 (td, 4H, -CH_2_NH-, J = 6.6, 6.6 Hz), 3.66 (m, 16H, -OCH_2_CH_2_O-(CH_2_CH_2_O)_4_-CH_2_CH_2_O-), 3.70 (t, 4H, -OCH_2_CH_2_O-(CH_2_CH_2_O)_4_-CH_2_CH_2_O-, J = 4.8 Hz), 4.09 (m, 4H, -NHCOOCH_2_CH_2_-), 4.23 (t, 4H, -OCH_2_CH_2_O-(CH_2_CH_2_O)_4_-CH_2_CH_2_O-, J = 4.8 Hz), 4.45-4.72 (br, 2H, -NHCOO-). ^13^C NMR (125 MHz, CDCl_3_): δ = 25.0 (-OOCCH_2_CH_2_-), 25.9 (-NHCOOCH_2_CH_2_-), 26.8 (-CH_2_-(CH_2_)_2_-NH-), 29.2 (-OOC-(CH_2_)_2_-CH_2_-), 29.3 (-OOC-(CH_2_)_3_-CH_2_-, -CH_2_-(CH_2_)_3_-NH-), 29.5 (-OOC-(CH_2_)_4_-(CH_2_)_3_-), 30.1 (-CH_2_CH_2_NH-), 34.4 (-OOCCH_2_-), 41.1 (-CH_2_NH-), 63.5 (-NHCOOCH_2_-), 64.5 (-OCH_2_CH_2_O-(CH_2_CH_2_O)_4_-CH_2_CH_2_O-), 69.4 (-OCH_2_CH_2_O- (CH_2_CH_2_O)_4_-CH_2_CH_2_O-), 70.7 (-OCH_2_CH_2_OCH_2_CH_2_O-(CH_2_CH_2_O)_2_-OCH_2_CH_2_CH_2_CH_2_O-), 70.8 (-OCH_2_CH_2_OCH_2_CH_2_O-(CH_2_CH_2_O)_2_-OCH_2_CH_2_CH_2_CH_2_O-), 156.8 (-NHCOOCH_2_-), 174.0 (-OOCCH_2_-). Elemental analysis (%) calcd for C_42_H_78_N_2_O_13_ (819.07): C, 61.47; H, 9.53; N, 3.46; found C, 61.59; H, 9.60; N, 3.42.

CEU(12): ^1^H NMR (300 MHz, CDCl_3_): δ = 1.28 (m, 44H, -(CH_2_)_7_-(CH_2_)_2_-NH-, -O-(CH_2_)_2_-(CH_2_)_8_-), 1.48 (m, 4H, -CH_2_CH_2_NH-), 1.63 (m, 8H, -OOCCH_2_CH_2_CH_2_-, -O-CH_2_CH_2_CH_2_-), 1.69 (m, 4H, -NHCOOCH_2_-(CH_2_)_2_-CH_2_COO-), 2.30 (t, 4H, -OOCCH_2_CH_2_-, J = 7.5 Hz), 3.16 (td, 4H, -CH_2_NH-, J = 6.6, 6.6 Hz), 4.07 (m, 8H, -NHCOOCH_2_CH_2_-, -OCH_2_CH_2_-), 4.46-4.70 (br, 2H, -NHCOO-). ^13^C NMR (125 MHz, CDCl_3_): δ = 25.2 (-OOCCH_2_CH_2_-), 25.9 (-NHCOOCH_2_CH_2_-), 26.1 (-O-(CH_2_)_2_-CH_2_-), 26.8 (-CH_2_-(CH_2_)_2_-NH-), 28.8 (-OCH_2_CH_2_-), 29.2 (-OOC-(CH_2_)_2_-CH_2_-), 29.3 (-OOC-(CH_2_)_3_-CH_2_-, -CH_2_-(CH_2_)_3_-NH-, -O-(CH_2_)_3_-CH_2_-), 29.5 (-OOC-(CH_2_)_4_-(CH_2_)_3_-, O-(CH_2_)_4_-(CH_2_)_2_-), 30.1 (-CH_2_CH_2_NH-), 34.6 (-OOCCH_2_-), 41.1 (-CH_2_NH-), 64.5 (-NHCOOCH_2_-, -OCH_2_CH_2_-), 156.8 (-NHCOOCH_2_-), 174.1 (-OOCCH_2_-). Elemental analysis (%) calcd for C_42_H_78_N_2_O_8_ (739.08): C, 68.25; H, 10.64; N, 3.79; found C, 67.95; H, 10.53; N, 3.69.

## 3. Results and Discussion

### 3.1. Synthesis and Characterization

Two novel types of polyurethanes containing ester linkages, namely a diol-diacid-type poly(ester-ether-urethane) (PEEU) and poly(ester-urethane) (PEU), and a hydroxy acid type poly(ester-diurethane) (PEDU), were designed and synthesized by a green method that avoided the use of diisocyanate (Scheme I and Scheme II). The urethane linkage was formed by the reaction of amine and phenyl carbonate, while ester linkages were periodically introduced into the main chain of the polymer by lipase to afford efficient chemical recycling segments.

First, tetramethylene bis(phenyl carbonate) (**1**) was prepared for the synthesis of the urethane linkage. Conventionally, **1** is produced by the reaction of phenyl chloroformate and butane-1,4-diol in the presence of a base catalyst [15,16]. In spite of the diisocyanate-free method for the synthesis of the urethane linkage, however, phenyl chloroformate is produced by the reaction of phenol and phosgene. Therefore, in order to avoid the use of materials derived from phosgene, a new route to **1** was carried out by the reaction of diphenyl carbonate (17 mmol) and butane-1,4-diol (0.85 mmol) with CALB in toluene solution. An excess amount of diphenyl carbonate was required to minimize oligomer formation. Next, the diurethane-containing dicarboxylic acid (**2**) was prepared by the reaction of **1** and sodium 12-aminododecanoate in chloroform/methanol. The urethane bond formed quickly without any catalyst.

The direct polycondensation of **2** and diol was carried out using CALB in 1,2-dimethoxybenzene as a solvent at 100 °C for 48 h. In the absence of CALB or in the presence of thermally deactivated CALB, no polycondensation of **2** and diol was observed. These results indicate that CALB is the active catalyst for the polycondensation process. Table 1 shows the polymerization conditions and some analytical data of the polymers.

**Figure 1:**
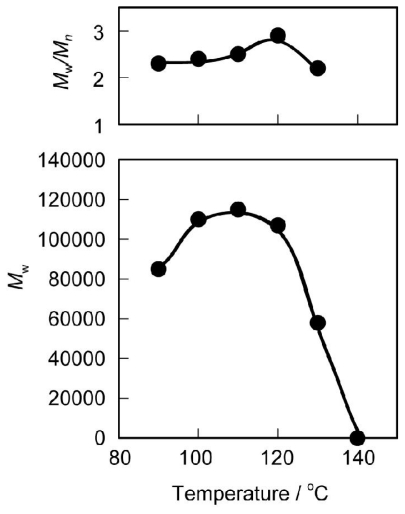
Effect of temperature on *M*_w_ and *M*_w_/*M*_n_ of PEEU(6). Reaction conditions: **2** (11.4 mg, 0.02 mmol), hexaethylene glycol (5.6 mg, 0.02 mmol), 1,2-dimethoxybenzene (0.057 mL), CALB (4.6 mg), 48 h.

The polymerization behavior of **2** with various diols was similar, irrespective of the oxyethylene or the methylene chain length, such that the *M*_w_s of the polymers were around 100,000 g·mol^−1^. The yields were 80–90% after purification by reprecipitation. Figure 1 shows the effect of temperature on *M*_w_ and *M*_w_/*M*_n_ of PEEU (6), prepared by the polycondensation of **2** and hexaethylene glycol. The *M*_w_ values of the polymer increased with reaction temperature from 90 to 100 °C, and were almost constant at around 110,000 g·mol^−1^ at temperatures between 100 and 120 °C. Reaction temperatures over 100 °C are higher than that used in aqueous solutions. However, it has been reported that under relatively dry conditions, enzymes, such as lipases, proteases and esterases, remain catalytically active at temperatures between 90–120 °C [17–20]. With temperatures over 120 °C, the *M*_w_ value gradually decreased due to thermal deactivation of the enzyme, and at 140 °C practically no polymer was produced. Therefore, further studies were carried out at 100 °C.

**Figure 2:**
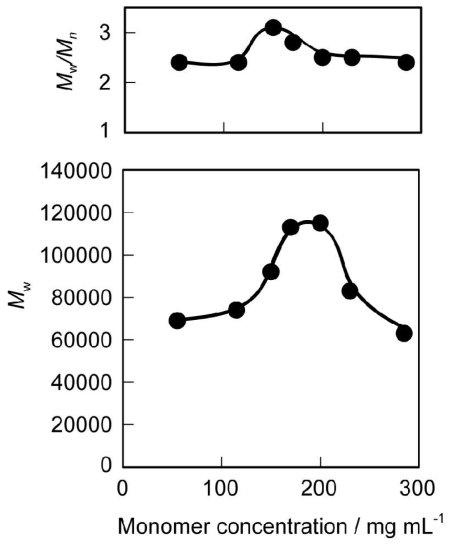
Effect of monomer concentration of **2** on *M*_w_ and *M*_w_/*M*_n_ of PEEU(6) at 100 °C. Reaction conditions: **2** (11.4 mg), hexaethylene glycol (5.6 mg), 1,2-dimethoxybenzene, CALB (4.6 mg), 48 h.

**Figure 3:**
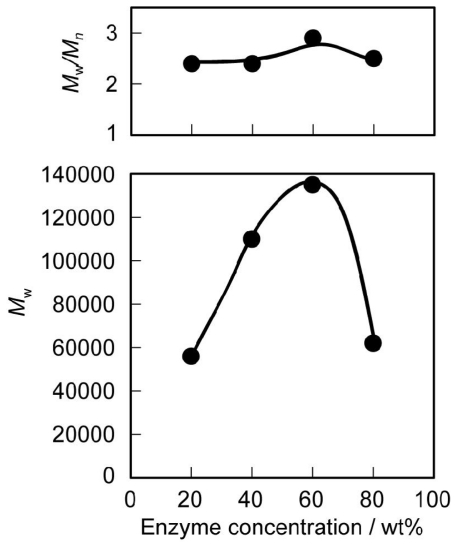
Effect of enzyme concentration (relative to **2**) on *M*_w_ and *M*_w_/*M*_n_ of PEEU(6) at 100 °C. Reaction conditions: **2** (11.4 mg, 0.02 mmol), hexaethylene glycol (5.6 mg, 0.02 mmol), 1,2-dimethoxybenzene (0.057 mL), CALB, 48 h.

**Figure 4:**
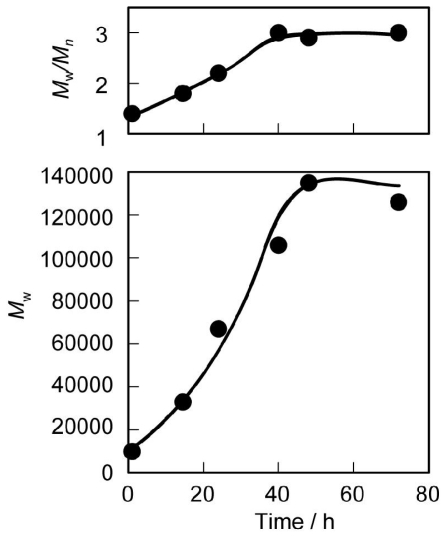
Time course of *M*_w_ and *M*_w_/*M*_n_ of PEEU(6) at 100 °C. Reaction conditions: **2** (11.4 mg, 0.02 mmol), hexaethylene glycol (5.6 mg, 0.02 mmol), 1,2-dimethoxybenzene (0.057 mL), CALB (6.8 mg).

The substrate concentration is the decisive factor for polymerization and depolymerization. In general, in a more concentrated solution, the equilibrium shifts toward polymerization. Figure 2 shows the effects of monomer concentration of **2** on *M*_w_ and *M*_w_/*M*_n_ of the polymer in 1,2-dimethoxybenzene solution. Polymerization occurred quickly and a high *M*_w_ value was produced at a monomer concentration around 200 mg·mL^−1^. The *M*_w_ of the polymer decreased with monomer concentrations higher than 200 mg·mL^−1^ due to the solidification of the polymerization system. The enzyme concentration also affected the *M*_w_ value of the polymer. Figure 3 shows the effect of enzyme concentration (relative to **2**) on *M*_w_ and *M*_w_/*M*_n_ of the polymer. Although CALB was immobilized on an acrylic resin, a relatively large amount of immobilized lipase was required for rapid polycondensation. The highest *M*_w_ was obtained for the polymerization at a lipase concentration of 60 wt%. However, the *M*_w_ of the polymer decreased with higher amounts of enzyme due to the increasing amount of enzyme containing water, which was responsible for both initiation and termination of the polymerization. Based on these results, further studies were carried out at 60 wt% lipase concentration. The *M*_w_ and *M*_w_/*M*_n_ values increased gradually with time. Figure 4 shows the time course for the polycondensation of **2** and hexaethylene glycol. *M*_w_ and *M*_w_/*M*_n_ values increased gradually by transesterification and reached equilibrium after 48 h.

In order to produce a polyurethane with a higher *T*_m_ compared to that of diol-diacid type PEEU(x) and PEU(y), a hydroxy acid type polyurethane (PEDU) with a higher content of urethane moieties was prepared. Diurethane containing hydroxy acid (**4**) was prepared by the reaction of **1** with 12-amino-1-dodecanol, followed by the reaction with sodium 12-aminododecanoate. It was found that **4** polymerized readily by CALB in anisole to produce PEDU with high *M*_w_ (*M*_w_ = 109,000 g·mol^−1^, Table 1).

The mechanism for the proposed lipase-catalyzed polymerization of hydroxy acid is basically the same as that for lactones [21,22]. Scheme III shows the proposed mechanism for lipase-catalyzed polycondensation of hydroxy acid-type monomer as a typical example. The initiation step is the formation of acyl-enzyme intermediate (EAM) via an enzyme-monomer complex. The propagation starts with nucleophilic attack of the EAM by the hydroxy group of the monomer, followed by successive nucleophilic attacks on the EAM by the terminal hydroxy group of the growing oligomer chain.

### 3.2. Thermal and Mechanical Properties of the Polyurethanes

Some thermal properties of PEEU(x), PEU(y) and PEDU were measured using DSC, and the results are summarized in Table 2. *T*_g_ could not be obtained by DSC analysis under the test conditions in this report. In Table 2, PDEU, a poly(diester-urethane) with an unsymmetrical molecular structure, previously reported [13], is shown as a reference (entry 7). PEEU(x), PEU(y) and PEDU are polymers with homogenous molecular structures, since **2** and **4** contain a symmetrical diurethane moiety. Due to the symmetrical structure of the polymers, PEEU(x), PEU(y) and PEDU showed higher *T*_m_, Young’s modulus and tensile strength compared to the previously reported poly(diester-urethane) (PDEU) [13].

**Figure A1:**
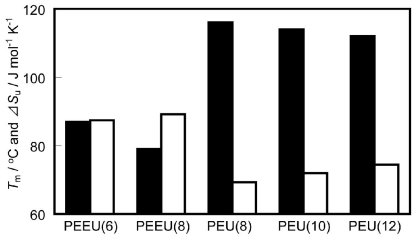
Thermal properties of PEEU(x) and PEU(y). *T*_m_ (■), Δ*S*_u_ (□).

Fusion enthalpy (Δ*H*_u_) was also determined by DSC measurements and the fusion entropy (Δ*S*_u_) was calculated from the *T*_m_ and Δ*H*_u_ values for PEEU(x) and PEU(y). The Δ*H*_u_ and Δ*S*_u_ values for PEEU(x) were 31.4–31.5 (kJ·mol^−1^) and 87.4–89.2 (J·mol^−1^·K^−1^), and for PEU(y) were 26.9–28.7 (kJ·mol^−1^) and 69.3–74.4 (J·mol^−1^·K^−1^), respectively (Appendix Figure A1). Ether-containing polymers [PEEU(x)] tended to show a lower *T*_m_ when compared to the corresponding methylene chain polymers [PEU(y)]. Since PEEU(x) had a higher Δ*S*_u_ (a parameter related to chain flexibility: an increase in Δ*S*_u_ indicates that the polymer is more flexible [23]) than PEU(y), the lower *T*_m_ was due to the greater flexibility of the ether moiety in the polymer chain (entries 1–5). The *T*_m_ was also affected by the content of the urethane moiety. *T*_m_ increased with the increasing urethane content, thus PEDU showed the highest *T*_m_ value among the poly(ester-urethane)s synthesized in this study.

PEEU(x) showed relatively lower values of Young’s modulus, elongation at break and tensile strength among the tested poly(ester-urethane)s. This is partially due to the flexibility of the ether moiety [24,25]. By extending the ether moiety (oxyethylene chain), the polymer became softer, but weaker. In contrast, PEU(y) and PEDU containing no ether moieties were harder and tougher than PEEU(x). It appeared that PEU(y) and PEDU were more similar to linear low-density polyethylene (LLDPE) [26–28] than some thermoplastic elastomers based on diphenylmethane diisocyanate (MDI) and polyols [29].

The static water contact angle is one of the factors affected by the polymer structure. The static water contact angle was measured by dropping distilled water on a glass slide coated with the polymers. The uncoated glass slide was used for the control (47.2°). PEEU(x) showed the lowest water contact angle (entries 1 and 2), indicating that the polymer surface is the most hydrophilic among the tested polymers. Since the composition of the urethane moiety is the same for PEEU(x) and PEU(y), the hydrophilicity is partially due to the ether moiety in the soft segment [24]. On the other hand, the water contact angles of poly(ester-urethane)s without the ether moiety were almost the same, between 83.8–85.9°, despite the length of the soft segment or the chemical structure of the polyurethane (entries 3–7).

### 3.3. Chemical Recycling of PEEU and PEU

**Figure 5:**
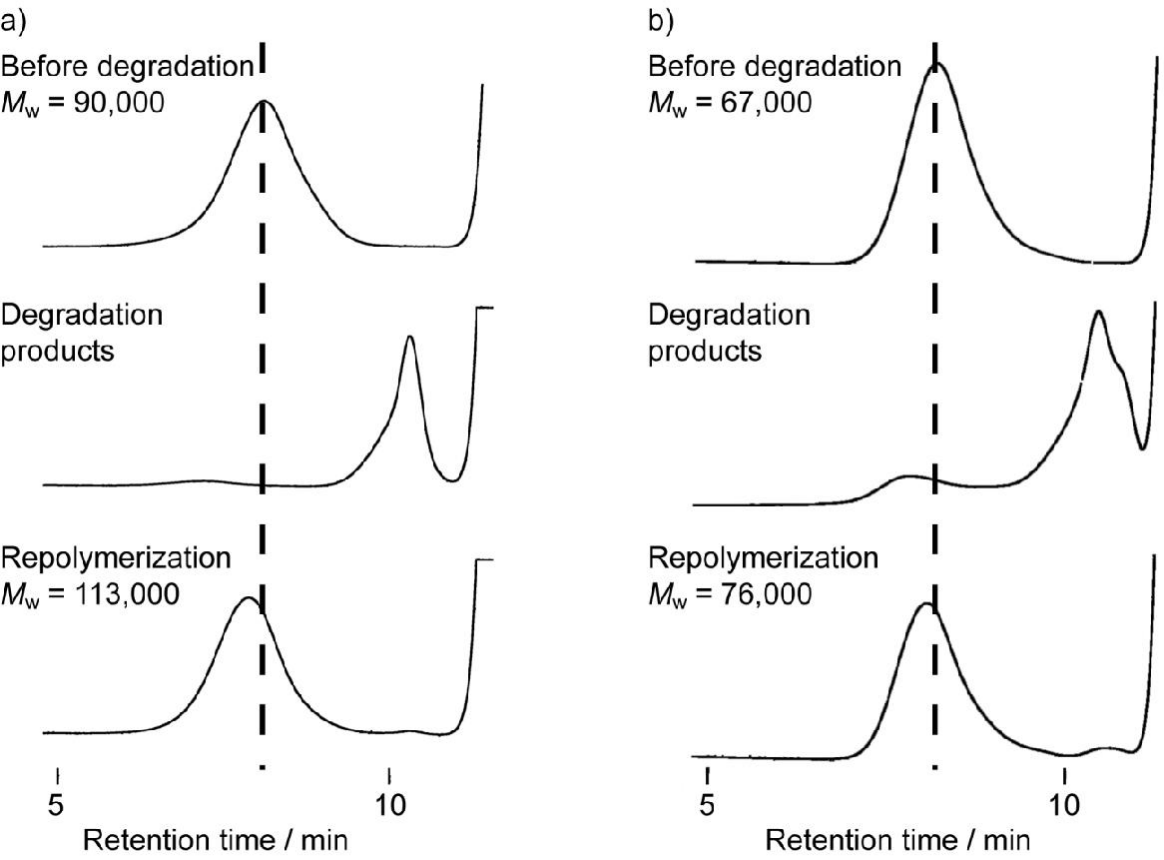
SEC profiles for PEEU(6) (**a**) and PEU(12) (**b**), their degradation products, and the repolymerization of the degradation product.

**Figure 6:**
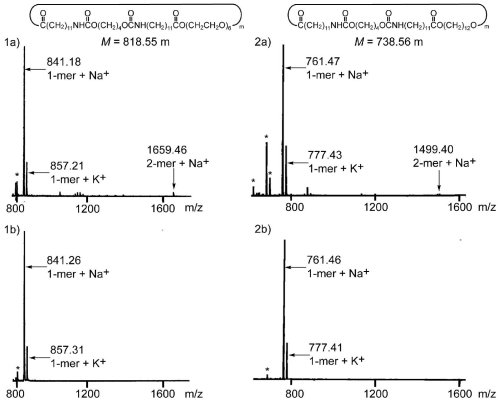
MALDI-TOF mass spectra of CEEU(6) before (**1a**) and after (**1b**) purification, and CEU(12) before (**2a**) and after (**2b**) purification. Reaction conditions: (**1a**) and (**2a**) 110 °C, PEEU(6) or PEU(12) (60.0 mg), anisole (20 mL), CALB (120.0 mg), 72 h; (**1b**) and (**2b**) purification by preparative SFC using scCO_2_ and ethanol. The asterisk (*) indicates the matrix.

**Figure A2:**
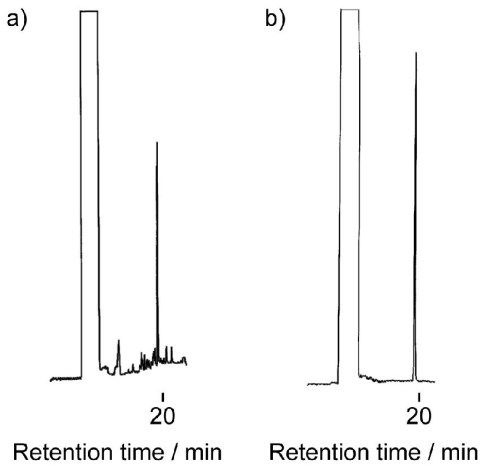
SFC profiles of CEEU(6) before (a) and after (b) purification.

**Figure A3:**
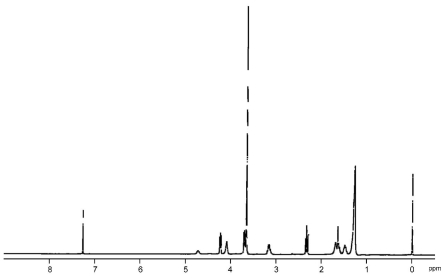
^1^H NMR spectrum of CEEU(6) in DMSO-*d_6_*.

The chemical recycling of diol-diacid-type PEEU and PEU was evaluated by their degradation into cyclic oligomers using a lipase in organic solvent. The concentration of the substrate is the decisive factor in the depolymerization, which is accelerated under dilute conditions (Scheme I). As a typical example, PEEU(6) was enzymatically degraded in anisole at 110 °C for 72 h in dilute conditions (3 mg·mL^−1^). The polymer degraded quickly to afford oligomers and no unreacted polymer was detected by SEC (Figure 5a). Figure 6 (1a) shows the MALDI-TOF mass spectrum of the crude degradation products. Molecular masses (*m/z*) of [818.55 m + Na^+^] and [818.55m + K^+^] corresponded to the structure of cyclic(ester-ether-urethane) (6) [CEEU(6)] oligomer. The cyclic monomer [m = 1 in Figure 6 (1a)] and a smaller amount of the cyclic dimer (m = 2) were the main components. The linear ester-ether-urethane oligomer, with a molecular mass 18 units higher than that of the corresponding cyclic oligomer fraction, was barely detected. In order to verify the cyclic structure, the crude product was purified by reprecipitation using chloroform (good solvent) and ethanol (poor solvent). The ethanol soluble part was collected and fractionated by using a preparative SFC with scCO_2_ and ethanol to obtain the molecularly pure CEEU(6) monomer (Appendix Figure A2). Figure 6 (1b) shows the MALDI-TOF mass spectrum of the isolated CEEU(6) monomer. The cyclic structure of the monomer was also confirmed by the absence in the ^1^H NMR spectra of peaks at δ = 2.15 ppm corresponding to the terminal C*H_2_*COOH moiety of **2** and at δ = 3.60 and 3.73 ppm corresponding to the terminal OC*H_2_*C*H_2_*OH moiety of the hexaethylene glycol moiety (Appendix Figure A3). The produced CEEU(6) monomer was repolymerized by CALB in a concentrated 1,2-dimethoxybenzene solution to produce PEEU(6), having similar *M*_w_ values as the initial polymer (Figure 5a). The chemical recycling of PEU(12) was also carried out by the same procedure as described for PEEU(6) (Figure 5b). These results confirm the chemical recyclability of PEEU(6) and PEU(12) by lipase.

## 4. Conclusions

High-molecular-weight poly(ester-ether-urethane)s, poly(ester-urethane)s and poly(ester-diurethane)s were produced by the lipase-catalyzed polymerization of urethane-containing diacids and polyethylene glycols, urethane-containing diacids and α,ω-diols, and urethane-containing hydroxy acids, respectively. The urethane linkage was readily created by the reaction of phenyl carbonate and amino compounds in high yields. Polyurethanes containing periodically introduced ester bonds were readily depolymerized into cyclic oligomers by lipase in a dilute solution, which were readily repolymerized into high-molecular-weight polyurethanes by the same lipase as in the chemical recycling process. The poly(ester-urethane)s tested in this study exhibited *T*_m_s between 79 and 132 °C, a Young’s modulus between 56 and 147 MPa, an elongation at break between 404 and 860% and a tensile strength between 13 and 35 MPa.

## Figures and Tables

**Scheme I:**
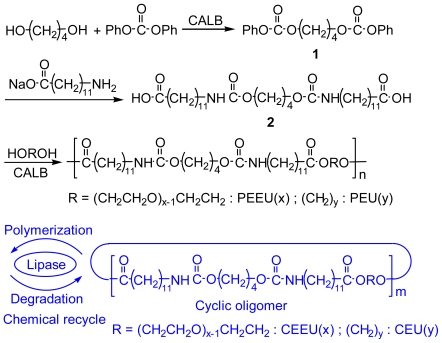
Preparation and chemical recycling of diol-diacid-type PEEU and PEU using an enzyme.

**Scheme II:**
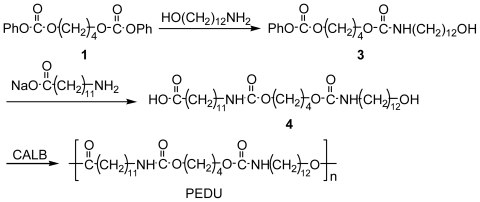
Preparation of hydroxy acid type PEDU using an enzyme.

**Scheme III:**
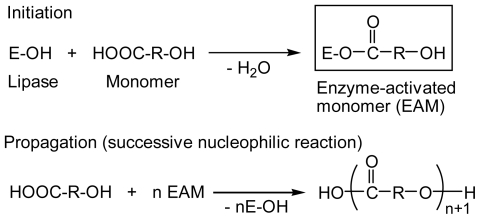
Proposed mechanism for lipase-catalyzed polymerization of hydroxy acid-type monomer.

**Table 1:** Preparation of poly(ester-urethane)s using CALB.

Polymer	Polymerization^a^				Polymer			
	Monomer 1	Monomer 2^b^	Solvent^c^	Temp./°C	Purification^d^	*M*_w_/g·mol^−1^	*M*_w_/*M*_n_	Yield/%
PEEU(6)	**2**	HEG	DMB	100	A	136,000	2.9	78.9
PEEU(8)	**2**	OEG	DMB	100	A	102,000	2.5	81.9
PEU(8)	**2**	1,8-OD	DMB	100	B	100,000	2.4	88.0
PEU(10)	**2**	1,10-DD	DMB	100	B	94,000	2.1	87.8
PEU(12)	**2**	1,12-DoD	DMB	100	B	101,000	2.6	86.3
PEDU	**4**	---	anisole	110	C	109,000	1.6	90.9

a Equimolar mass of monomer **1** and monomer **2** was polymerized using CALB for 48h;

b HEG: hexaethylene glycol; OEG: octaethylene glycol; 1,8-OD: octane-1,8-diol; 1,10-DD: decane-1,10-diol; 1,12-DoD: dodecane-1,12-diol;

c DMB: 1,2-dimethoxybenzene;

d Reprecipitation. A: DMF/methanol; B: CHCl_3_/methanol-*n*-hexane; C: CHCl_3_/DMF.

**Table 2:** Thermal and mechanical properties of polyurethanes.

Entry	Polymer	*T*_g_/°C	*T*_c_/°C	*T*_m_/°C	Young’sm odulus/MPa b	Elongation at break /% b	Tensile strength/MPa b	Water contact angle/° c
1	PEEU(6)	-	70 a	87	87	527	17.8	75.6
2	PEEU(8)	-	60 a	79	56	404	13.2	71.7
3	PEU(8)	-	87	116	90	677	26.8	83.8
4	PEU(10)	-	85	114	87	738	28.1	84.0
5	PEU(12)	-	86	112	91	860	33.2	84.2
6	PEDU	-	123	132	147	639	35.1	85.9
7	PDEU [13]	−33	-	42	29	1,383	11.1	85.0
8	LLDPE [26–28]	−128	111	126	129	1,104	19.1	102.0
9	TPU_MDI-PEA-EG_ [29]	-	-	195–248	-	425	47	-

a *T*_c_ was collected in a cooling after a first heating;

b Cross-head speed: 30 mm·min^−1^;

c Control: slide glass; 47.2°.

## Appendix NMR Analysis

PEEU(8): ^1^H NMR (300 MHz, CDCl_3_): δ = 1.27 (m, 28H, -(C*H**_2_*)_7_-(CH_2_)_2_-NH-), 1.48 (m, 4H, -C*H**_2_*CH_2_NH-), 1.61 (m, 4H, -OOCCH_2_C*H**_2_*CH_2_-), 1.70 (m, 4H, -NHCOOCH_2_-(C*H**_2_*)_2_-CH_2_COO-), 2.32 (t, 4H, -OOCC*H**_2_*CH_2_-, *J* = 7.5 Hz), 3.15 (td, 4H, -C*H**_2_*NH-, *J* = 6.6, 6.6 Hz), 3.65 (m, 24H, -OCH_2_CH_2_O-(C*H**_2_*C*H**_2_*O)_6_-CH_2_CH_2_O-), 3.69 (t, 4H, -OCH_2_C*H**_2_*O-(CH_2_CH_2_O)_6_-C*H**_2_*CH_2_O-, *J* = 4.8 Hz), 4.07 (m, 4H, -NHCOOC*H**_2_*CH_2_-), 4.22 (t, 4H, -OC*H**_2_*CH_2_O-(CH_2_CH_2_O)_6_-CH_2_C*H**_2_*O-, *J* = 4.8 Hz), 4.48–4.71 (br, 2H, -N*H*COO-).

PEU(8): ^1^H NMR (300 MHz, CDCl_3_): δ = 1.27, 1.32 (m, 36H, -(C*H**_2_*)_7_-(CH_2_)_2_-NH-, -O-(CH_2_)_2_- (C*H**_2_*)_4_-), 1.48 (m, 4H, -C*H**_2_*CH_2_NH-), 1.61 (m, 8H, -OOCCH_2_C*H**_2_*CH_2_-, -O-CH_2_C*H**_2_*CH_2_-), 1.68 (m, 4H, -NHCOOCH_2_-(C*H**_2_*)_2_-CH_2_COO-), 2.33 (t, 4H, -OOCC*H**_2_*CH_2_-, *J* = 7.5 Hz), 3.15 (td, 4H, -C*H**_2_*NH-, *J* = 6.6, 6.6 Hz), 4.05 (m, 8H, -NHCOOC*H**_2_*CH_2_-, -OC*H**_2_*CH_2_-), 4.50–4.71 (br, 2H, -N*H*COO-).

PEU(10): ^1^H NMR (300 MHz, CDCl_3_): δ = 1.27, 1.29 (m, 40H, -(C*H**_2_*)_7_-(CH_2_)_2_-NH-, -O-(CH_2_)_2_- (C*H**_2_*)_6_-), 1.48 (m, 4H, -C*H**_2_*CH_2_NH-), 1.61 (m, 8H, -OOCCH_2_C*H**_2_*CH_2_-, -O-CH_2_C*H**_2_*CH_2_-), 1.67 (m, 4H, -NHCOOCH_2_-(C*H**_2_*)_2_-CH_2_COO-), 2.29 (t, 4H, -OOCC*H**_2_*CH_2_-, *J* = 7.5 Hz), 3.15 (td, 4H, -C*H**_2_*NH-, *J* = 6.6, 6.6 Hz), 4.05 (m, 8H, -NHCOOC*H**_2_*CH_2_-, -OC*H**_2_*CH_2_-), 4.49–4.70 (br, 2H, -N*H*COO-).

## References

[1] Kihara N, Endo T (1993). Synthesis and properties of poly(hydroxyurethane)s. J Polym Sci A Polym Chem.

[2] Rokicki G, Piotrowska A (2002). A new route to polyurethanes from ethylene carbonate, diamines and diols. Polymer.

[3] Neffgen S, Keul H, Höcker H (1997). Cationic ring-opening polymerization of trimethylene urethane: A mechanistic study. Macromolecules.

[4] Kusan J, Keul H, Höcker H (2001). New routes to [n]-polyurethanes. [3]-polyurethane: Synthesis, characterization, and polymerization-depolymerization equilibrium. Macromol Symp.

[5] Kusan J, Keul H, Höcker H (2001). Cationic ring-opening polymerization of tetramethylene urethane. Macromolecules.

[6] Helou M, Carpentier J-F, Guillaume SM (2011). Poly(carbonate-urethane): An isocyanate-free procedure from alpha,omega-di(cyclic carbonate) telechelic poly(trimethylenecarbonate)s. Green Chem.

[7] Campbell GA, Melunch WC (1977). Polyurethane waste disposal process development: Amine recovery. J Appl Polym Sci.

[8] Simioni F, Bisello S, Tavan M (1983). Polyol recovery from rigid polyurethane waste. Cell Polym.

[9] Simioni F, Modesti M, Rienzi SA (1987). Polyol recovery from elastomer polyurethane waste. Cell Polym.

[10] Simioni F, Modesti M, Tavan M (1991). Controlled degradation of polyurethane for recycling. Mater Eng.

[11] Kanaya K, Takahashi S (1994). Decomposition of polyurethane foams by alkanolamines. J Appl Polym Sci.

[12] Matsumura S (2006). Enzymatic synthesis of polyesters via ring-opening polymerization. Adv Polym Sci.

[13] Yanagishita Y, Kato M, Toshima K, Matsumura S (2008). Chemoenzymatic synthesis and chemical recycling of sustainable polyurethanes. Chem Sus Chem.

[14] Soeda Y, Toshima K, Matsumura S (2005). Synthesis and chemical recycling of novel poly (ester-urethane)s using an enzyme. Macromol Biosci.

[15] Al-Hamouz OCS, Sweileh BA, Al-Salah HA (2006). Synthesis and characterization of polycarbonates by melt-phase interchange reactions with alkylene and arylene diphenyl dicarbonates. J Appl Polym Sci.

[16] Sweileh BA, A-Hiari YM, Aiedeh KM (2008). A new, nonphosgene route to poly(bisphenol a carbonate) by melt-phase interchange reactions of alkylene diphenyl dicarbonates with bisphenol A. J Appl Polym Sci.

[17] Volkin DB, Staubli A, Langer R, Klibanov AM (1991). Enzyme thermoinactivation in anhydrous organic-solvents. Biotech Bioeng.

[18] Parvaresh F, Robert H, Thomas D, Legoy MD (1992). Gas-phase transesterification reactions catalyzed by lipolytic enzymes. Biotech Bioeng.

[19] Turner NA, Duchateau DB, Vulfson EN (1995). Effect of hydration on thermostability of serine esterases. Biotech Lett.

[20] Turner NA, Vulfson EN (2000). At what temperature can enzymes maintain their catalytic activity?. Enzyme Microbiol Technol.

[21] Uyama H, Takeya K, Kobayashi S (1995). Enzymatic ring-opening polymerization of lactones to polyesters by lipase catalyst—Unusually high reativity of macrolides. Bull Chem Soc Jpn.

[22] MacDonald RT, Pulapura SK, Svirkin YY, Gross RA, Kaplan DL, Akkara J, Swift G, Wolk S (1995). Enzyme-catalyzed ε-caprolactone ring-opening polymerization. Macromolecules.

[23] Min BM, Son TW, Jo WH, Choi SG (1992). Thermal stability of polyacrylonitrile in the melt formed by hydration. J Appl Polym Sci.

[24] Sarkar D, Yang J, Lopina ST (2008). Structure-property relationship of l-tyrosine-based polyurethanes for biomaterial applications. J Appl Polym Sci.

[25] Pierce BF, Brown AH, Sheares VV (2008). Thermoplastic poly(ester urethane)s with novel soft segments. Macromolecules.

[26] Stehling FC, Mandelkern L (1970). The glass temperature of linear polyethylene. Macromolecules.

[27] Luyt AS, Hato MJ (2005). Thermal and mechanical properties of linear low-density polyethylene/ low-density polyethylene/wax ternary blends. J Appl Polym Sci.

[28] Yuan Z, Chen H, Zhang J, Zhao D, Liu Y, Zhou X, Li S, Shi P, Tang J, Chen X (2008). Preparation and characterization of self-cleaning stable superhydrophobic linear low-density polyethylene. Sci Technol Adv Mater.

[29] Pricariu C, Olley RH, Caraculacu AA, Bassett DC, Martin C (2003). The effect of hard segment ordering in copolyurethane elastomers obtained by using simultaneously two types of diisocyanates. Polymer.

